# Diversity of Concerns in Recovery after a Nuclear Accident: A Perspective from Fukushima

**DOI:** 10.3390/ijerph15020350

**Published:** 2018-02-16

**Authors:** Akiko Sato, Yuliya Lyamzina

**Affiliations:** 1 United Nations University Institute for the Advanced Study of Sustainability, Tokyo 150–8925, Japan; 2 Radiation Medical Science Center for the Fukushima Health Management Survey, Fukushima Medical University, Fukushima 960–1295, Japan; lyamzina@fmu.ac.jp

**Keywords:** Fukushima Daiichi Nuclear Power Station accident, nuclear accident, nuclear disaster, disaster management, post-accident operation, radiation risk, risk perception, risk communication, psychological distress

## Abstract

Since the 2011 Fukushima nuclear accident, tremendous resources have been devoted to recovery, and the Japanese Government is gradually lifting evacuation orders. However, public concerns remain prevalent, affecting some people’s return to a normal life and threatening their well-being. This study reviews government reports, academic papers, newspaper articles and conference presentations with the aim of obtaining a better understanding of issues which relate to radiation concerns in the recovery process in the aftermath of the accident. It looks extensively at: (1) the current status of the post-accident operations and existing radiation issues in Fukushima, and (2) approaches taken to engage the public during recovery from five previous comparable nuclear and radiological events: Three Mile Island, Buenos Aires (RA-2 facility), Chernobyl, Goiânia and Tokai-mura. The findings indicate that the limitations and emerging challenges of the current recovery operations cause concerns about radiation exposure in various aspects of day-to-day life. Past experiences suggest that long-term management that take a holistic and cohesive approach is critical for restoration of sustainable livelihoods and for social re-integration. Not only actual risks but also public perceptions of risks should be carefully assessed and addressed in the process of environmental remediation.

## 1. Introduction

“*The Fukushima nuclear accident abruptly deprived our peaceful everyday lives*.”

“*Fukushima is receiving lesser attention. But the disaster is not over, and the torment remains*.”

These were common statements made to the first author of this article (Sato) during interviews with affected residents and civil organizations in Fukushima in 2015. Although over six years have passed since the meltdown of nuclear fuel rods and several major explosions at the Fukushima Daiichi Nuclear Power Station (FDNPS) of the Tokyo Electric Power Company (TEPCO), the accident continues to cast a shadow over Fukushima and neighbouring regions.

The nuclear accident emitted a large amount of radioactive microparticles, such as iodine-131 and caesium-137. The International Atomic Energy Agency (IAEA) [1] estimates that 100–400 petabecquerels (PBq) of iodine-131 and 7–20 PBq of caesium-137 were released into the atmosphere, and 10–20 PBq of iodine-131 and 1–6 PBq of caesium-137 went directly into the North Pacific Ocean, acknowledging variations in estimates. Aoyama et al. [2] analysed the amount of caesium dispersed from the FDNPS reactors and estimated that a total of 19–24 PBq of caesium-137, which has a half-life of approximately 30 years and will pose long-term consequences for Fukushima, was discharged into the environment, including 15–20 PBq into the atmosphere, of which 12–15 PBq entered the ocean, which received a total of 15–18 PBq.

Even though the estimated amounts of iodine-131 and caesium-137 discharged from the FDNPS were roughly 10% and 20% of those in the 1986 Chernobyl accident, the Fukushima accident was classified as a Level 7 “major accident”, which is the most serious level, on the International Nuclear and Radiological Event Scale (INES), together with the Chernobyl accident, for the significance of its effects on people and the environment [3,4]. Of around 600 workers at Chernobyl in the early morning of 26 April 1986, 134 developed acute radiation syndrome due to exposure to a high radiation dose (0.8–16 grays (Gy) [5]. Of those, 28 died in the first 4 months, and another 19 died from various causes by 2006, although many of those deaths were unlikely to be directly associated with radiation exposure. Most of the more than 500,000 workers involved in the recovery operations were exposed to 0.02–0.5 Gy between 1986 and 1990, receiving an overall average dose of 0.12 Gy. Some post-disaster studies [5,6,7,8] discuss increased risks of certain illnesses in the workers, such as leukaemia, but further evidence and clarifications are necessary in order to confirm the health effects of radiation exposure.

At Fukushima, however, there were no radiation-related casualties during or immediately after the accident. Among nearly 50,000 workers involved in the reactor decommissioning and decontamination operations at FDNPS (as of March 2016) and over 30 million workers engaged in clean-up activities in affected areas (as of March 2017), it was not until October 2015 that a case of leukaemia in a nuclear worker (diagnosed in January 2014) was officially acknowledged as the first “workplace accident” connected to the accident [9,10,11,12]. Nowadays, there is wide recognition among local and international experts and institutions that the magnitude of the Fukushima accident is not as severe as the Chernobyl case [13,14,15].

Since the accident, three rounds of thyroid ultrasound examinations of those who were still in utero or aged up to 18 years at the time of the accident have been conducted as part of the Fukushima Health Management Survey (the 3rd round is still in progress as of January 2018), which was established to monitor the health status of Fukushima residents, evaluate impacts of long-term low-dose exposure, and promote residents’ overall well-being through the provision of medical services and health counselling programs when needed. Over 300,000 children and adolescents of the target population of approximately 368,000 (82%) underwent the first round of screening (preliminary baseline screening) between October 2011 and March 2014 with an extension up to April 2015 for those who had not participated previously. Around 271,000 children and adolescents of about 381,000 (71%) underwent the second round (full-scale thyroid screening) between April 2014 and March 2016. About 138,000 had participated in the third round as of June 2017. Among them, screening identified 154 confirmed malignancy cases (of which 101 cases were identified in the first round) [16,17,18]. Although there is no precise pre-accident epidemiological data of the population based on the same screening procedure, the data do not differ meaningfully from data collected from other prefectures by an adaptation of the same methodology [19,20,21]. Discussions are still in progress; however, on account of the age and time of onset (thyroid cancer cases were identified in older age groups and earlier than the cases reported after the Chernobyl accident), low levels of radiation exposure, a diet rich in natural iodine (which should have some protective effect), and the Government’s prompt food control measures, dominant perspectives consider that cases identified during the first few rounds of thyroid ultrasound examination after the Fukushima accident are unlikely to be due to radiation exposure, but are existing cases or recently emerged cases detected as a consequence of the large-scale, high-resolution screening [19,22,23].

Furthermore, currently available monitoring data reported by governmental institutions, independent agencies and experts show a considerable decline in environmental radioactivity levels since March 2011 [22,24]. Present radiation levels in many parts of Fukushima are comparable to or even lower than natural radiation background in other locations inside and outside Japan [24,25]. Whole-body-counter evaluations of internal radiocaesium contamination have also demonstrated that a vast majority of Fukushima residents fell below detection levels, reinforcing the United Nations Scientific Committee on the Effects of Atomic Radiation’s (UNSCEAR’s) perspective of very small health risks due to direct exposure from the accident [26,27].

As radiation levels decline and infrastructure is rebuilt, the Government is gradually reopening municipalities in the evacuation zone. Large parts of the affected areas had already been reopened by the end of fiscal year (FY) 2016. The Government is aiming at terminating its evacuation order for some parts of even the most affected areas by the end of FY 2021, where access is not currently permitted [28]. Still, as of July 2017, 57,538 people remain evacuated, although this is now roughly one-third of the peak in May 2012 (164,865) [24]. Many evacuees are reluctant to return for various reasons, such as a lack of employment opportunities; housing problems, including the repair or reconstruction of abandoned houses; insufficient infrastructure, such as schools, hospitals and shops; and persistent concerns about radiation [29]. Despite the above data and experts’ views, public anxiety over the health effects of radiation is still grave, resulting in, among other effects, serious school bullying of children displaced from affected areas [30].

At the same time, a number of people have already returned to their homes in places where evacuation orders have been withdrawn and are re-starting their lives. However, even among returnees and those who were never required to leave, radiation concerns remain strong [31,32]. Persistent and somewhat polarized concerns, in fact, have directly or indirectly led to family separations and social divisions, and are strongly associated with psychological distress and post-traumatic stress disorder, along with other concerns such as the loss of livelihood and financial challenges [33]. Furthermore, lifestyle-style related illnesses have become more significant after the accident. Increases in both mental and physical ill-health are considerably affecting overall well-being and the recovery process in Fukushima [33,34,35,36,37]. Post-accident data suggest that mental health issues are particularly severe in Fukushima, even compared with other areas affected by the Great East Japan Earthquake and tsunami, because of the unfamiliarity of such large-scale technological disaster and challenges created by long-lasting radiological substances released from FDNPS [34]. Multiple complexities of the nuclear accident, involving long-term environmental contamination and social problems, are expected to take longer to repair than the structural damage caused by the natural disasters [38,39]. The local people’s comments introduced in the beginning of this paper and the cited reports show that there is a clear gap between experts’ views and public perceptions about the accident and the current radiation situation, and that Fukushima still has a long way to go.

Japan is now going through a critical period for the sustainable recovery from the accident. The above-described situations in Fukushima underscore the importance and urgency of obtaining a good understanding of issues which cause persistent concerns among the public in the recovery process for the sustainable restoration of livelihoods and social integration. For these reasons, we carried out this study: (1) to investigate and outline the current status and progress of the post-accident operations, the key challenges and the existing radiation issues in Fukushima, and (2) to review and gain insights from past approaches taken for environmental remediation and post-accident recovery in order to further understand the challenges in Fukushima and common issues in the recovery process in the aftermath of major nuclear accidents.

## 2. Methods

After the Japanese Government’s announcement at the end of 2016 of its plan to accelerate disaster recovery measures and to begin reopening the most affected areas in Fukushima [28], we conducted a situation analysis between June–November 2017 gathering information on the recovery operations and associated socio-environmental conditions surrounding local people. We extensively reviewed government documents and websites, academic papers, technical reports, newspaper articles and conference presentations on the topic. We focused review on the latest progress made in 2016–2017 which, according to the government, was the initial two years of the “Reconstruction and Revitalization Period” (2016–2020) following the “Intensive Reconstruction Period” (2011–2015) [38].

To fulfill the second objective of this study we completed a narrative comparison of the Fukushima case. We selected and studied five major nuclear or radiological accidents of the past few decades, namely (1) the 1979 accident at the Three Mile Island Nuclear Power Station in the USA, (2) the 1983 accident at the RA-2 facility of the National Atomic Energy Commission Constituyentes Atomic Center near Buenos Aires, Argentina, (3) the 1986 accident at the Chernobyl Nuclear Power Station in Ukraine, (4) the 1987 accident in Goiânia, Brazil, and (5) the 1999 accident at the nuclear fuel conversion facility of JCO in Tokai-mura, Japan. Selection was based on the significance of their impacts on the environment or society. We investigated the main response and recovery measures of these accidents after reviewing the estimated magnitude of the events and their health outcomes. We predominantly searched websites and technical reports of international organizations and respective national governments, as well as academic papers.

Science Direct, JSTOR and Google Scholar were used to search scholarly articles with various key terms, such as Fukushima and the other five nuclear accidents selected for this study, as well as, “radiation concerns”, “recovery”, “environmental remediation” and “decontamination”. Targeted searches were performed to obtain materials from the Ministry of the Environment of Japan, the Reconstruction Agency of Japan, the Fukushima Prefectural Government, the Fukushima Medical University (which led the Fukushima Health Management Survey), TEPCO, IAEA, UNSCEAR and the World Health Organization. Some books were also included in this review as they contain data which reinforce and supplement the governments’ materials and academic papers, and because they provide new information on the circumstances and impacts of Fukushima and the other past events, as well as the countermeasures taken for public safety and environmental remediation. 

## 3. Results

### 3.1. Progress in Post-Accident Operations

Under the Government’s policy of reducing individual radiation exposure and avoiding further accidents at the FDNPS, the clean-up of contaminated areas and the decommissioning of the damaged reactors have become the main objectives of the recovery. The legal framework, strategies and implementation plans of these activities were formed relatively quickly after the accident, and enormous human and financial resources have been dedicated every year towards recovery [40,41,42].

#### 3.1.1. Progress in Decontamination of Residential and Agricultural Areas

“*There can be no recovery or restoration of the disaster-stricken areas without rapid removal and treatment of disaster waste*.”

—Goshi Hosono, Minister of the Environment, Japan (2012) [43]

With this recognition, clean-up activities have been implemented in the contaminated towns where the additional annual exposure dose was greater than 1 millisievert (mSv) [44]. The Ministry of the Environment had allocated about JPY 2.6 × 10^12^ (equivalent to roughly USD 24 × 10^9^) until March 2017, and it is estimated that over 30,000,000 workers have been involved in the decontamination operations. Except in the most contaminated area, where the estimated cumulative dose exceeded 50 mSv per year as of March 2012, all planned clean-up activities related to homes, roads and public facilities are nearly completed in Fukushima and other affected prefectures, covering more than 588,000 houses, 41,000 hectares (ha) of farmland and 10,000 has of forest [11,45].

Environmental measurements have shown some effectiveness of decontamination in reducing radiation levels, although the effects vary widely according to the severity of contamination and geographical conditions [46,47]. Relative to pre-decontamination data, on average, air dose rates were reduced by 56% in residential areas, 58% in farmland and 23% in forest, immediately after decontamination [11]. Six-month post-operation data suggest further declines. The Government has since earmarked JPY 30 × 10^9^ (≈ USD 270 × 10^6^) in the FY 2017 budget for further decontamination activities in the most affected areas [48].

The large-scale decontamination work is creating enormous amounts of radioactive waste. In the past 6 years, approximately 16,000,000 cubic meters (m^3^) of contaminated soil and other materials has been removed. The Government directed that the materials be placed in “interim” storage within Fukushima for eventual final disposal outside the region. The construction of interim storage facilities around the FDNPS began in 2015. By October 2017, approximately 438,000 m^3^ of contaminated waste had been transferred there. The Government plans to increase transfer to 12,500,000 m^3^ in FY 2020, for final disposal outside the prefecture in about 30 years’ time [11,45]. In contrast, the volume of low-level radioactive waste is being reduced by incineration; for instance, the amount of stored sewage sludge was reduced from 75,700 tonnes (t) in September 2013 to 5600 t in April 2017 [24].

In addition, research facilities such as the Centre for Environmental Creation and the Environmental Radiation Centre were established in Fukushima to evaluate and improve decontamination technologies, closely monitor radiation situations in the prefecture and share information with residents and others [24].

#### 3.1.2. Progress in Decommissioning Fukushima Daiichi Nuclear Power Station

The accident at the FDNPS was complex. In short, the Great East Japan Earthquake and subsequent massive tsunami on 11 March 2011 caused the station’s coolant system to fail, which led to fuel meltdowns in Units 1 to 3, where reactors were operating. Hydrogen explosions at Units 1 and 3 blew off the tops of the reactor buildings. Unit 2 experienced major damage to the pressure-suppression system connected to the reactor vessel. Units 4, 5 and 6 were already shut down for routine inspection at the time of the accident, but the Unit 4 reactor still experienced a major explosion, possibly due to a hydrogen leak from the adjacent Unit 3 [49].

Decommissioning of the damaged FDNPS is an urgent priority to secure the safety of residents and others. Despite the complexity of the damage, several important advances have been made in the past six years. One notable example is the removal of all fuel rods from the spent-fuel pool of Unit 4 by the end of 2014. In addition, TEPCO crews removed 20 t of highly radioactive rubble from Unit 3 in August 2015, and finished removing the remaining ceiling panels of Unit 1 by October 2015. Multiple advanced technologies, such as robots and drones, have been introduced to enable workers to investigate dangerous areas [50,51]. For example, TEPCO and scientists have used the detection of subatomic particles called muons to generate images of the plant interiors, and found in September 2015 that the accident had melted over 70% of the fuel in Unit 2 [52]. Robot operation managed to investigate the submerged parts of Unit 3 in July 2017 and identified signs of molten fuel debris [53].

### 3.2. Challenges in Post-Accident Operations

Despite this extensive work, the current situation in Fukushima demonstrates that recovery from a major nuclear accident is not a linear process, but is a long, messy process of uncertainty which uncovers further problems with its progress. This subsection summarizes current key challenges in decontamination and the decommissioning of the FDNPS.

#### 3.2.1. Challenges in Decontamination of Residential and Agricultural Areas

Since the general procedure of “decontamination” work involves removing the top 5 cm of soil, along with leaves, branches and other materials, whether or not they are contaminated (generally < 2 mSv), it is producing an enormous volume of low-level radioactive waste. It is estimated that the final amount will reach as much as 22,000,000 m^3^ after incineration [45]. Over 800 “temporary” storage sites have been created in local communities across Fukushima to hold millions of black plastic bags filled with the soil and other materials until they can be removed off site. The construction of temporary storage facilities, however, is slower than the rate of waste accumulation, and temporary storage sites established by local government do not allow full regional coverage. Consequently, a large amount of low-level radioactive waste remains at nearly 150,000 collection sites (as of December 2016), including home gardens and school grounds [24]. Only 60% of the total 1600 ha intended for interim storage facilities is yet secured as of September 2017, and delays are notable [45]. On top of that, very little progress has been made in identifying a site and measures for final disposal [54].

Despite some improvements in incineration, as decontamination is still in progress, reducing the volume of waste will remain a critical challenge for Fukushima for many years ahead. The volume of waste could be reduced significantly by high-pressure washing to separate radioactive materials and incinerating the debris, and by reusing soil with contamination below 8000 Bq/kg for construction works such as road pavement and coastal breakwaters, according to the Government, but soil with contamination of 5000 Bq/kg may require about 170 years for the radiation level to go down to the safety standard of 100 Bq/kg stipulated before the accident [55]. With strong concerns from some experts and local people, the Government has been reviewing the approach and seeking measures for safety assurance [56,57].

In addition, existing decontamination techniques are incapable of instantly reducing the radiation level to pre-accident levels (<1 mSv/year). Furthermore, some areas remain untouched and are not included in current decontamination plans. Those areas, such as forests, lakes, and rivers, are largely uninhabited and have limited human activities. The present policy for decontamination was developed in consideration of several elements, such as estimates of radiation exposure dose rates for local people and the effectiveness of clean-up activities at lowering doses. Priority was given to farms to help the region and population to return to normal as soon as possible by revitalizing agriculture. Over 70% of the land area in Fukushima prefecture is covered by forest, and clean-up work has generally been performed only within 20 m of homes [44,58].

#### 3.2.2. Challenges in Fukushima Daiichi Nuclear Power Station Decommissioning

Despite some progress in the past 6 years, decommissioning is still at a very early stage of an estimated 30- to 40-year process owing to the complexity of removing extremely hot and severely contaminated fuel debris from the damaged reactors, as well as to difficulties in processing and disposing of the large amount of radioactive waste generated during the process [59].

Many problems have already been reported. For example, TEPCO’s investigation of reactor buildings using remote-control robots has been a painful trial-and-error process [60]. In early 2017, TEPCO managed to examine the reactor vessel in Unit 2, but the situation there was far worse than expected: possible melted fuel spread extensively within the vessel and a few wide holes in the grating beneath the reactor. Tokyo Electric Power Company recorded an estimated 650 Sv per hour, which can kill a human being in seconds [61]. Data from the muon scan indicated that nearly all of the fuel rods in Unit 1 and most of those in Unit 3 have melted through the reactor vessel [62,63,64]. Furthermore, the team discovered a dislodged concrete reactor cover, which was intended to prevent radiation leaks, in Unit 1, and damage to exhaust pipes, especially in Units 1 and 2. To handle these issues first, the Government and TEPCO decided to delay the removal of spent fuel from the reactors from FY 2020 to FY 2023, and to delay the finalization of methods to take out and dispose of the melted fuel rods for another year [64,65].

Another critical struggle for TEPCO is the management of the rapidly accumulating highly contaminated water used to maintain the reactor cooling systems, as well as the influx of groundwater into the basements of the damaged reactors. There have been multiple serious water leaks. In 2013, for example, high levels of radioactive isotopes, including tritium, were found in groundwater near Units 1 and 2. It is estimated that between 20 and 40 terabequerels (TBq) of tritium leaked towards the ocean between May 2011 and July 2013 [66]. Tokyo Electric Power Company reported another leak of 300 t of highly contaminated water from a tank in August 2013, and another leak of contaminated rainwater into the ocean though a drainage ditch in February 2015 [67,68]. Tokyo Electric Power Company constructed impermeable underground walls along the coastline and additional sub-drains. In August 2017, it announced that it had started the final work to complete the installation of “frozen soil walls” to stop groundwater pouring into the damaged reactors and to prevent contaminated water from escaping into the soil and ocean. However, multiple difficulties and consequent significant delays have been reported. Tokyo Electric Power Company claims that the rate of accumulation of contaminated water has reduced from 300 t/day to 130 t/day [69]. Nevertheless, there is a view [70] that the improvement is due mainly to other countermeasures, such as the sub-drains, and that the contribution of the frozen soil walls is limited. A careful evaluation is required to determine the effectiveness of the walls.

Tokyo Electric Power Company established a facility to remove radioactive strontium and other elements from contaminated water, but it cannot remove tritium. The total amount of water treated has gone up to about 800,000 t by July 2017 [71]. The Government and TEPCO deem that tritium-contaminated water is not harmful even if it is released into the ocean. This perspective has raised serious concerns among residents, including fishermen, over health effects, as well as possible noxious rumours about seafood safety, which can result in significant economic damage [71]. The issue of contaminated water is still contentious.

Because of these problems, future decommissioning involves many large uncertainties. In fact, the operations are expected to become more complicated and more difficult as decommissioning progresses, requiring more innovative technologies entailing additional enormous resources. It is estimated that the total cost for the decommissioning will go beyond the initial estimate of 2 × 10^12^ JPY [72], and it may take much longer than expected.

### 3.3. Factors Linked to Current Radiation Concerns in Fukushima

One fundamental issue in Fukushima is that the accident posed considerable impacts on both humans and the natural environment, and clean-up activities have been carried out in many neighbourhoods. Consequently, people’s concerns about radiation can arise from any aspect of life in relation to remaining contaminants, additional contamination from non-clean-up areas, and possible radiation exposure in the process of radioactive waste management, such as from the sites where contaminated waste is currently stored and publicly accessible, or in the future during the transportation of the accumulated waste to interim storage facilities. People could be exposed to radioactive materials in Fukushima in two main ways:

1. Being exposed to radioactive materials outside the body (*external exposure*), such as those deposited on the ground.
2. Taking in radioactive materials in water or food, such as in wild foods in contaminated areas (*internal exposure*). Some people are worried about the contamination of the water supply systems and food chain [73,74,75,76].

Table 1 summarizes potential radiation sources which could influence public perceptions and the level of anxiety over long-term radiation exposure and possible health risks.

In Fukushima, some people continue to have direct involvement in forests. Many people cannot totally divorce themselves from Nature. Radioactive materials in forests can enter the groundwater that some households rely on for drinking, cooking, washing and bathing. Radioactive materials attached to soil, leaves and other surfaces in forests could also be dispersed by rain, snow and wind. In addition to the remaining radioactivity after the clean-up is finished, possible contamination from those untouched areas might increase concerns over low-dose exposure [76]. There are also non-negligible psychological impacts of having dozens of bags filled with contaminated materials in view or passing by on trucks [33].

In September 2015, torrential rain swept away bags from some temporary storage sites in Fukushima and neighbouring prefectures. The Government announced that no substantial contamination was detected, but the incident shows the unstable conditions after the 2011 accident [77]. In addition, since the disaster, Fukushima has experienced several earthquakes of magnitude 5 or larger. Each earthquake caused great concerns among the public about possible impacts on the FDNPS, although no critical safety-related incidents have been reported [78,79].

It is important to point out that government and private institutions as well as experts monitor radiation. Empirical studies demonstrate that the conservative method which the Government uses actually overestimates individual external doses [80,81]. This indicates that radiation-related health risks from the accident are lower than initial estimates. Furthermore, crop and livestock products and seafood from Fukushima are inspected constantly for contamination by local institutions before being brought to the market. The radiation levels of nearly all food items, with a few rare exceptions of wild plants and mushrooms and inland fishery products, are below international safety standards and even lower than stringent government regulatory dose limits [73,82,83,84]. However, some people remain afraid of existing and further contamination in view of the remaining non-decontaminated areas, the transport of contaminated materials, major delays in handling decontamination wastes, the considerable challenges in the FDNPS decommissioning, and recurrent natural hazards, and are keeping an anxious watch on the recovery process.

### 3.4. Insights from Past Nuclear and Radiological Events

The likelihood of accidents and the magnitude of their possible consequences have been major concerns both for the public and experts, and have been the subject of considerable, often heated debate since the first nuclear facility was established [85]. In fact, several radiation accidents with varying impacts have been recorded. For instance, the UNSCEAR 2008 report [5] lists over 30 notable accidents at nuclear facilities, including those involving nuclear weapons programs, as well as 80 accidents at other industrial facilities, such as those related to radioactive sources kept at the facilities. Of the five events selected for this review (the 1979 Three Mile Island accident, the 1983 Buenos Aires RA-2 facility accident, the 1986 Chernobyl accident, the 1987 Goiânia accident, and the 1999 Tokai-mura accident), some had significant impacts on the environment and people, and others caused severe damage and contamination to the facilities but released little radioactivity outside the facilities, but nevertheless triggered considerable fears in local communities. These accidents have been rated as Level 4 (“accident with local consequences”), Level 5 (“accident with wider consequences”) or Level 7 (“major accident”) on the INES [86]. Table 2 presents a summary of the accidents, focusing on the estimated magnitude of the events and their health outcomes, and the main response and recovery measures.

Table 2 shows first that accidents can occur at any time anywhere. Furthermore, these events highlight several distinct types of accidents: (1) possibly catastrophic and fatal, (2) potentially trans-boundary, (3) often human-induced and highly technical, and (4) possibly posing long-term impacts on people and the environment and requiring extensive, long-term operations for recovery.

Most victims of many of the past accidents were plant workers who were on site when the accident happened, or were first responders, such as fire fighters at Chernobyl. In 1987 in Goiânia, victims were family members or employees of a scrapyard owner who unknowingly bought the hazardous radiotherapy source stolen from an abandoned hospital. Except for those who died in the explosion at Chernobyl, these victims were exposed to a lethal dose of radiation in a short period of time, and died of severe radiation sickness in the first few months or days [90,92,96,98]. Some people survived high doses for various reasons, such as dose fractionation, which allows the body to repair tissue damage caused by radiation. Some accidents, such as the 1999 accident at the JCO facility, left survivors who experienced serious radiation sickness and even developed bone marrow failure, and yet who recovered after rigorous medical treatment [100].

Importantly, most past accidents had no or few direct casualties, despite the number of people exposed to radiation. However, in the aftermath of the Chernobyl accident, a substantial increase in some illnesses, including thyroid cancer and leukaemia, was identified. Nearly 5000 confirmed cases of thyroid cancer were reported by 2002 among children and adolescents aged up to 18 at the time of the accident in Belarus, Ukraine and Russia [93]. In total, 11,000 cases had been identified by 2016 in this subpopulation in those countries [101]. There are diverse views among experts concerning the increase of those illnesses, and significant challenges are involved in proving causality and quantifying long-term health effects in epidemiological studies.

Second, the past accidents demonstrate that radioactive contamination can be widespread, sometimes going beyond national borders. Wind and rain, for example, can carry long-lived radioactive materials in any direction and significantly affect people and the environment over large areas. Radiation fallout from Chernobyl and subsequent exposure affected millions of people in Europe outside Belarus, Ukraine and Russia, albeit with much smaller average doses. Izrael and colleagues [102] estimated that for caesium-137 alone, of the total release of 8 × 10^16^ Bq over Europe, nearly 34% fell in Belarus, 24% in Russia, 20% in Ukraine, 4% in Sweden, 4% in Finland, 3% in Bulgaria and 3% in Austria. 8 × 10^16^ Bq Radioactive contaminants can spread via other media as well. It is arguable that contaminants in the Pacific Ocean from the 2011 accident in Fukushima reached the shores of North America, although the level is considered non-hazardous [103]. Following the 1987 accident in Goiânia, radioactive substances spread among the public through unprotected handling of the source, as well as though daily activities in the home [96].

Third, all of the accidents reviewed involved technological failures, more specifically, human-induced errors made in designing, testing or maintaining nuclear facilities and their functions, or in processing or handling nuclear or other radioactive materials, in addition to the violation of safety procedures. These failures generally stemmed from inadequate individual capacities and substandard safety systems for plant operations [87,90,92,96,98]. In addition, nuclear and radiologic technologies demand specialized, intricate skills which require many years of advanced training. One operational mistake could lead to catastrophic consequences. For many laypersons, radiation is an unfamiliar and highly technical subject. Moreover, it is undetectable to the unaided senses. This nature can induce great fear among the public and make communicating associated risks extremely important in the promotion of risk avoidance or reduction behaviours, and of public understanding of remediation and waste management operations after accidents. At the same time, the nature of radiation makes risk communication complex and challenging [104].

Fourth, long-term radiation contamination posed by nuclear or radiological accidents may cause prolonged or even permanent changes in day-to-day lives of affected people. In Goiânia, the area where the collected radioactive waste is stored must remain out of bounds for over 300 years [97]. In Chernobyl, a huge concrete shield was erected over the decaying reactor in 2016 to prevent further release of radioactive substances for at least 100 years [95]. Yet highly contaminated land is still strictly closed, and agricultural and industrial activities are prohibited. Workers engaged in the decommissioning of the reactors or radiation monitoring also work under strict conditions, such as living outside the area, keeping set shift lengths, being monitored for radiation exposure and undergoing periodic health checks [105]. It is unknown whether the area will ever be habitable again. These situations have forced local people to abandon their livelihoods, homes and land, although a few hundred mostly senior citizens have returned. Persistent psychological or mental health problems, including post-traumatic stress disorder, are significant among people affected by the Chernobyl accident, and an increased suicide rate among clean-up workers has been reported [93,106]. Even in less affected areas, notable disruption intended to minimize or prevent radiation exposure has occurred, such as changes to diet [107]. These consequences show that nuclear events can have considerable psychological and social impacts.

Given the significance of the physical, psychological and emotional impacts of radiation-related accidents, interactive community-based approaches have been introduced in the aftermath of these accidents. For instance, the Citizen Radiation Monitoring Program, begun after the Three Mile Island accident, which involved radiation monitoring of residents living nearby, enabled lay citizens to acquire basic knowledge of radiation, to measure and obtain precise data concerning their environment, and to reduce some of the fear derived from their unfamiliarity with radiation science [108]. Such a participatory approach was also applied after the Chernobyl accident: notable examples include the ETHOS project (1996–2001) and the CORE (COoperation for REhabilitation of living conditions in the Chernobyl-affected areas of Belarus) project (2003–2009) in Belarus, intended to strengthen safety measures and improve the quality of living conditions (such as health, diet and social activities) as a core part of the post-accident management strategy [109,110]. The projects promoted the establishment of a community-based monitoring system to enable inhabitants to acquire comprehensive data and information to cope with the conditions. They were designed to address the differences in individual lifestyles which may affect the level of radiation exposure. They also aimed at fostering a radiation protection culture by encouraging the active involvement of residents. These experiences suggest that citizen empowerment through community-based technical support is important for the sustainability of the projects’ positive contributions to the long process of recovery after a major nuclear accident.

## 4. Discussion

The long recovery process after a severe nuclear accident is a very complex issue requiring tremendous effort and resources. The Fukushima accident resulted in extensive impacts on both humans and the natural environment, and clean-up has been carried out a massive-scale in many neighbourhoods in Fukushima and other affected prefectures. Decommissioning of the FDNPS and remediation activities are still in progress. Although the Fukushima accident was classified as Level 7 on the IAEA INES scale, as was the 1986 Chernobyl accident, available data suggest that the overall releases of radioactive substances were much less than at Chernobyl. 

Since the accident, various post-accident measures have been taken, and notable progress has been made in the past six years. Owing to radioactive decay, weathering by rain and wind, and decontamination work, radiation levels have declined considerably, and the Japanese Government is withdrawing the evacuation orders. A good number of residents have already returned or are returning home. The process is still under way, and therefore the population size and estimated return rate are constantly changing. Furthermore, the return rate varies largely by municipalities. For instance, the evacuation order for Kawauchi village was lifted first among municipalities that were fully evacuated. Most of the village lies within 30 km of FDNPS; large parts of it were reopened in April 2012, and the remaining parts were reopened in October 2014 and June 2016. In Kawauchi, 2197 of 3038 residents (the number registered at the time of the accident), or 72%, have returned as of December 2017 [111]. Most of Naraha town lies within 20 km of FDNPS; it was the first town reopened in the designated “exclusion zone” (September 2015). Here, in contrast to Kawauchi, 2105 of 8011 residents, or 26%, have returned as of December 2017 [112].

In heavily contaminated areas, moreover, it is unrealistic to expect that recovery operations will be completed or that the areas will be fully reopened for many years to come, although decontamination work has started. Many unsolved problems remain, such as in the assessment of the situation inside the damaged reactors and the finalization of methods to remove and dispose of melted fuel rods, as well as in the management of the extremely large and still rapidly accumulating body of radioactive waste. Japan and Fukushima are facing and will continue to face considerable technical and financial challenges for many years in achieving safe and responsible recovery.

At the same time, this accident has reminded Japan and the rest of the world once again that nuclear accidents are not only a matter of dealing with radiation itself; they also have substantial social implications. Health problems caused by long-term displacement and consequent drastic lifestyle changes are much more significant than the direct effects of radiation. As well as other life challenges created by the Fukushima accident, the accident has had extensive emotional and psychological tolls on affected residents stemming from diverse and persistent concerns about radiation. These wider consequences clearly show that the recovery process requires a long-term, comprehensive approach for sustainable livelihood reconstruction and stabilization.

Past events, including that at Fukushima, provide key lessons in the management of long-term recovery from a major nuclear accident:

1. It is critical to monitor the different dimensions of the processes of post-accident remediation operations and their implications for affected residents. Approaching the social-psychological dimensions of an accident must be recognized as a priority alongside infrastructure rebuilding and environmental remediation. Not only risks of radiation but also other health risks should be carefully monitored and addressed.
2. Citizen participation in situation monitoring and evaluation and individual measurement of radiation should be encouraged and supported technically and financially. Open channels of communication among scientists and the public should be established. It is important to make comprehensive, coherent, easily understandable information on the recovery process available from sources which people are familiar with and have easy access to. Given the magnitude and persistence of adverse mental health effects which a nuclear accident can have on people, there is a strong need for careful consideration of perceived risks in addition to actual risks in stakeholder communication.
3. Continued efforts should be given to strengthening international networks and collaboration in the long-term process of recovery from a major technological accident. It is important to integrate lessons learned from past accidents into both international and national nuclear safety requirements and standards. Disaster management strategies should include the need for long-term governance of recovery, as well as concrete and practical approaches.

The list of past events, especially if we include less serious accidents and incidents, show that nuclear accidents are not rare events. The nuclear industry is still in the process of development, and nuclear safety is still not yet fully established. Substantial improvements in technology, human capacities and oversight mechanisms are required. It is also time for each country which has or considers building nuclear plants to assess the need for the plants in the first place with a comprehensive and thorough cost–benefit analysis. The possible human, environmental, economic and social costs of an accident should be fully considered. The Fukushima case clearly shows that there is still a need to develop further knowledge in radiation sciences, as well as in the management of nuclear accidents, such as more effective and efficient measures for environmental remediation and the decommissioning of damaged reactors based on careful cost-benefit analyses, and measures to alleviate radiation concerns and minimize overall health risks. It is, therefore, important to closely monitor the recovery process and deepen our understanding of the indirect impacts of nuclear accidents on the overall well-being of affected people, together with health issues directly attributable to radiation exposure. This will help governments and policy makers move towards more effective policies and management in consideration of people’s perceptions about their environment and the long, complex recovery process in their communities and how it affects their livelihoods. 

## 5. Conclusions

This study looks at the current status and progress of the post-accident operations in Fukushima and reviews the current key challenges including existing radiation issues. It underscores the continuing complexity of the environmental and social issues in the aftermath of the nuclear accident. Since the accident, there has been notable progress in the post-accident environmental remediation operations, which, along with naturally-occurring effects such as radioactive decay and weathering, has contributed to a significant decline in environmental radioactivity levels. It has also been found that radiation-related health risks from the accident are lower than initial estimates, which themselves showed limited risks to human health. However, the limitations and emerging challenges of the current recovery operations contribute to persistent day-to-day concerns about radiation exposure among local people. This study highlights that these issues are not unique to Fukushima nuclear accident. Experiences from the past events also demonstrate the significance of social-psychological challenges and suggest that the challenges would not be solved easily in a short time. Effective environmental remediation is vital for safety assurance, and it has to be cost-effective for a sustainable recovery. At the same time, a heavy focus solely on environmental remediation operations might divert attention from local people’s perceptions about radiation risks, which could delay and even make the recovery process more complex. Not only actual risks but also public perceptions of risks should also be carefully assessed and addressed. It is therefore critical to carry out a long-term risk management that is comprehensive and closely engages affected people for restoration of sustainable livelihoods and for social re-integration. The Fukushima nuclear event has reminded Japan and the world that nuclear accidents can happen anywhere at any time, and when they do, they may have very severe and complex consequences on both the environment and the society. Hence, there is a strong need to improve nuclear disaster risk reduction and management mechanisms. Such approach will help us be better prepared for any possible nuclear accidents in the future.

## Figures and Tables

**Table 1:** Potential sources, causes or risks of radiation exposure that may influence public perceptions and anxieties.

Residential and Farm Land			Forests & Other “Untouched” Areas	FDNPS
External exposure				
Radioactive waste	Temporary storage sites Interim storage facility Streets: transport to interim storage facility		Forests: soil, leaves, fallen leaves	Secondary incidence during reactor decommissioning and subsequent release of radioactive materials
Soil	Home gardens Agricultural land	Remaining radio­nuclides in residential areas		
Dwellings	Roofs, gutters			
Internal exposure				
Agricultural products			Groundwater and water from reservoirs for domestic and agricultural uses	Food or water contamination through: leakage of radioactive water into the environment, such as to the Pacific Ocean secondary incidents during reactor decommissioning
			Wild plants, animals and mushrooms Fishes from rivers and lakes Seafood	

**Table 2:** Summary of selected nuclear or radiological events in the past few decades.

Location	Three Mile Island [5,87,88,89]	RA-2 Buenos Aires [90,91]	Chernobyl [5,92,93,94,95]	Goiânia [96,97]	Tokai-mura [98,99,100]
Date	28 March 1979	23 September 1983	26 April 1986	13 September 1987	30 September 1999
INES *	5	4	7	5	4
Event description	Operational and system flaws led to malfunction of the reactor coolant systems and partial meltdown in the Unit 2 reactor	During the modification of core configuration in an experiment in RA-2 reactor of the Constituyentes Atomic Center, operational errors caused energy excursion	Deficiencies in reactor design and operational error during an evening safety test caused uncontrolled nuclear reactions, immense hydrogen explosion, fires and core meltdown	Improper dismantling and removal of radiation therapy device and radiation source left in an abandoned clinic resulted in public radiation exposure	Operational error in fuel processing at the nuclear fuel conversion plant resulted in nearly 20 h of uncontrolled self-sustaining nuclear chain reaction
Estimated amount of radiation released	~370 PBq (up to 480 PBq) of noble gases such as xenon and krypton were released, but less iodine-131 (around 550 GBq) and other harmful substances were released	The accident triggered an est. 3 × 10^17^ fissions. The excursion was about 10 MJ **	Released 14 EBq, containing 1.8 EBq of iodine-131 and 0.085 EBq of caesium-137 and other caesium radioisotopes (as of 26 April 1986); half of the total release was noble gases	The source contained 50.9 TBq in the form of caesium chloride (caesium-137)	During 2.5 × 10^18^ fissions, large doses of neutrons and gamma rays dispersed (est. ≥ 160 TBq of noble gases and 2 TBq of gaseous iodine released but remained in building)
Area of evacuation and number of people displaced	Advice for evacuation was given to pregnant women and pre-school-age children in an 8-km radius of the plant; additionally, 144,000 within 24 km and 195,000 within 32 km left home	No official evacuation order was issued to the public	Area within a 30-km+ radius became closed, and >330,000 were displaced (around 116,000 from the 30-km zone)	~200 who were living in the most contaminated area were temporarily displaced	161 living within a 350-m radius of the plant were evacuated, while those in a 10-km radius were told to stay indoors (orders were lifted 2 days later)
Direct health outcomes	No health problems which could be directly attributable to the accident were reported	Nearly ten people were exposed to radiation: one died (20 Gy *** of gamma rays and 17 Gy of neutrons) 2 days later	134 showed acute radiation syndrome (0.8–16 Gy); 28 died in 4 months; >530,000 recovery workers, evacuees and other inhabitants in contaminated areas were exposed; marked increases in some health defects and illnesses were reported, especially among those aged 0–20 years in 1986	>112,000 examined: 249 diagnosed as contaminated; 20 required intensive medical care; 4 died (est. dose of 4.5–6 Gy) in the first few months	>660 were evaluated: 3 experienced severe acute radiation syndrome, of whom 2 died (5.4 Gy of neutrons + 8.5 Gy of gamma rays, 12 weeks; 2.9 Gy of neutrons and 4.5 Gy of gamma rays, 7 months)
Countermeasures	Clean-up work on the damaged Unit 2 was conducted. The fuel was removed, and the reactor was permanently closed. Radioactive waste was transported off-site.	The reactor was decommissioned and dismantled in 1984–1989. The area has since been reopened for unrestricted use.	Mitigation and decontamination are done mainly at the facility and in the exclusion zone. The destroyed reactor was shielded by enclosing it in concrete. Access to many of the most affected areas remains prohibited.	The radiation source and contaminated sites were identified and cleaned up. The waste was buried to be kept in a repository for 300 years.	The nuclear reaction was stopped, and contaminated materials were shielded. The facility was closed permanently.

* International Nuclear Event Scale. ** Megajoules. *** Gray. **** 1 GBq (gigabecquerel) = 10^9^ Bq (becquerel), 1 TBq (terabecquerel) = 10^12^ Bq, 1 PBq (petabecquerel) = 10^15^ Bq, 1 EBq (exabecquerel) = 10^18^ Bq.
